# *QuickStats*: Age-Adjusted Colorectal Cancer Death Rates,* by State — United States, 2024

**DOI:** 10.15585/mmwr.mm7522a3

**Published:** 2026-06-11

**Figure:**
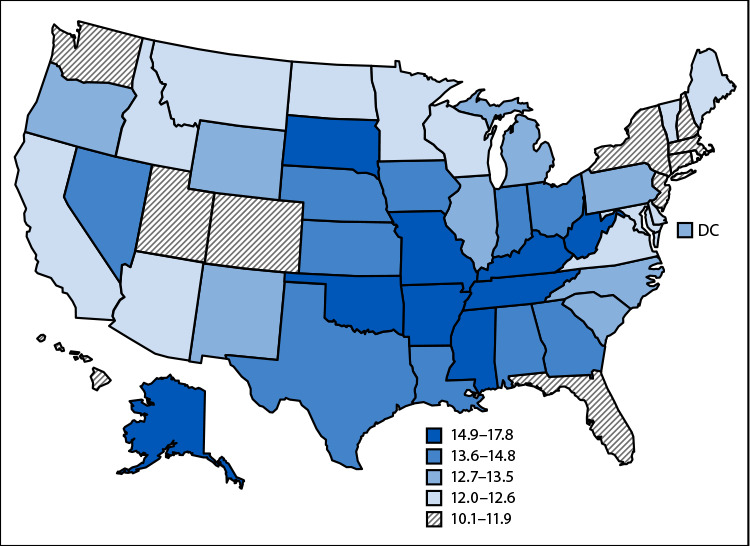
In 2024, the U.S. colorectal cancer death rate was 12.9 deaths per 100,000 standard population. Rates were generally lower in the Northeast and higher in the South. Colorectal cancer death rates were highest in Oklahoma (17.8) and lowest in Rhode Island (10.1).

**Supplementary Table:** https://stacks.cdc.gov/view/cdc/256552#tabs-3

**Source:** National Center for Health Statistics, National Vital Statistics System, Mortality Data, 2024. Underlying Cause of Death, 2018–2024, Single Race Request

For more information on this topic, CDC recommends the following link: Colorectal Cancer | CDC.

